# Older adults’ readiness to engage with eHealth patient education and self-care resources: a cross-sectional survey

**DOI:** 10.1186/s12913-018-2986-0

**Published:** 2018-03-27

**Authors:** Nancy P. Gordon, Mark C. Hornbrook

**Affiliations:** 1 Division of Research, Kaiser Permanente Northern California, Oakland, CA USA; 2 Center for Health Research, Kaiser Permanente Northwest Region, 3800 North Interstate Avenue, Portland, OR 97227 USA

**Keywords:** Digital divide, eHealth, eHealth literacy, Disparities, Race-ethnicity, Seniors, Internet use, Patient education

## Abstract

**Background:**

This study examined access to digital technologies, skills and experience, and preferences for using web-based and other digital technologies to obtain health information and advice among older adults in a large health plan. A primary aim was to assess the extent to which digital divides by race/ethnicity and age group might affect the ability of a large percentage of seniors, and especially those in vulnerable groups, to engage with online health information and advice modalities (eHIA) and mobile health (mHealth) monitoring tools.

**Methods:**

A mailed survey was conducted with age-sex stratified random samples of English-speaking non-Hispanic white, African-American/black (black), Hispanic/Latino (Latino), Filipino-American (Filipino), and Chinese-American (Chinese) Kaiser Permanente Northern California members who were aged 65–79 years. Respondent data were weighted to the study population for the cross-sectional analyses.

**Results:**

Older seniors and black, Latino, and Filipino seniors have less access to digital tools, less experience performing a variety of online tasks, and are less likely to believe that they would be capable of going online for health information and advice compared to younger and white Non-Hispanic seniors. Consequently, they are also less likely to be interested in using eHIA modalities.

**Conclusions:**

The same subgroups of seniors that have previously been shown to have higher prevalence of chronic conditions and greater difficulties with healthcare access are also less likely to adopt use of eHIA and mHealth monitoring technologies. At the patient population level, this digital divide is important to take into account when planning health information and chronic disease management programs. At the individual patient level, to provide good patient-centered care, it is important for providers to assess rather than assume digital access, eHealth skills, and preferences prior to recommending use of web-based resources and mHealth tools.

**Electronic supplementary material:**

The online version of this article (10.1186/s12913-018-2986-0) contains supplementary material, which is available to authorized users.

## Background

Health and healthcare-related information and patient education are increasingly being provided through web-based and email communications in addition to print and oral modalities, making information and advice about how to maintain or improve health and manage diseases more widely and freely accessible to all segments of the population. Numerous apps are available to provide ongoing monitoring of vital signs and support for chronic disease self-management and healthy lifestyle behaviors. *Healthy People 2020* includes an expanded set of goals regarding use of eHealth (“health communication strategies and health information technology to improve population health outcomes and health care quality and to achieve health equity”) [1]. However, lack of easy access to the digital tools that enable access to internet-based health education and advice (eHIA) and mobile device-based monitoring (mHealth) resources, skills in using these tools and resources, and preferences for how to obtain health information and advice may limit achievement of these *Healthy People* goals [2–4].

The segment of the U.S. population that will likely be most impacted by the shift toward use of eHealth and mHealth modalities for patient education and chronic disease management is adults aged ≥65 (seniors). In national surveys, U.S. seniors are significantly less likely than middle-aged adults to be going online [5, 6], and those not already online are unlikely to start in the future [5]. Among seniors, computer access and ability to use the internet have been shown to be lower among blacks and Hispanic/Latinos than among non-Hispanic whites [7–17], those aged ≥75 [5, 8, 10, 11, 13, 15–18], and those with lower incomes [8, 15] and lower educational attainment [7, 8, 10, 11, 13, 16–18].

Use of the internet for health-related purposes has been rapidly increasing. Most health plans and physician practices have patient portals that enable secure communications between patients and clinicians, as well as other functions such as viewing lab test results, ordering prescriptions, making appointments, and looking up medical history [19, 20], and health care organizations frequently have information about their physicians and services, downloadable forms, and patient education material on non-secure patient-facing websites. The internet has also become an important channel for people to obtain information about health conditions, drugs and other treatments, and providers of treatments; to search for results of medical research; to connect with people who have similar health-related issues through online communities and forums; and to download or purchase apps and medical supplies to help them monitor vital signs, manage chronic conditions, and lead a healthy lifestyle [9, 21–23]. However, older adults are less likely to be using the internet than other age groups for these purposes. A 2013 survey found that even among those who used the internet, seniors were significantly less likely than middle-aged adults to have gone online for health information in the past year [6]. A longitudinal survey of a national cohort of U.S. seniors (mean age 75) also found relatively low rates of use of digital technologies for health-related purposes in 2014 [24].

In recent years, study of the digital divide has expanded from a focus on identifying sociodemographic factors associated with access to the internet and digital devices to identifying factors associated with use of eHealth and mHealth resources among those who have access to these tools. For example, Horrigan describes “digital readiness” as having the digital skills necessary to perform different tasks using the internet, a belief in one’s ability to determine the trustworthiness of online or digitally-produced information and to safeguard personal information when going online, and the extent to which people currently use digital tools in the course of carrying out online tasks [25]. Similarly, eHealth literacy describes capacity to seek, find, understand, and appraise health information from electronic sources and apply the knowledge gained to addressing or solving a health problem [26–28]. Other factors being explored include trust in the internet [29] and attitudes and preferences for using digital technologies [30, 31].

To investigate the readiness of seniors to engage with these new digital platforms for health-related purposes, in late 2013–2014, we conducted a survey of older adults in Kaiser Permanente’s Northern California health plan about access to, skills and experience in using, and preference for using web- and mobile-based modalities. In an earlier publication [17], we drew on data from the survey to report on racial/ethnic and age-related differences in seniors’ ability to use the internet and email, perceived ability and willingness to perform selected health care-related tasks online, including completion of health questionnaires, preferences regarding emailed information, and engagement with the health plan’s patient portal. In this article, we focus on the digital divide in (1) perceived ability to use a variety of eHealth and mHealth resources, (2) recent experience performing online tasks relevant to patient education and self-care, including paying for products and services, and (3) preferences for using different eHealth modalities to obtain health information and advice. This study was approved by Kaiser Foundation Research Institute’s Institutional Review Board.

## Methods

### Setting and study population

This study was conducted with adult members aged 65–79 of the Kaiser Permanente Medical Care Program in Northern California (KPNC), an integrated healthcare delivery system serving over 2.5 million adults. The KPNC adult membership is similar to the insured adult population of Northern California with regard to sociodemographic and health characteristics [32]. KPNC has a comprehensive website (kp.org) that provides information that is accessible to both members and the public about the health plan and health-related topics, and a secure patient portal that is only available to health plan members. In 2013, approximately 419,000 (17.4%) KPNC members were aged 65 or older, and five racial/ethnic groups (non-Hispanic white, black, Hispanic/Latino, Filipino, and Chinese) accounted for approximately 95% of all non-limited English proficient health plan members in this age group.

#### Survey sample

We conducted the survey with a stratified random sample of 5420 KPNC members aged 65–79 who had a primary language of English in their electronic medical record. The initial sample included 1420 non-Hispanic white (white), 1500 African-American/black (black), 1500 Hispanic/Latino (Latino), 500 Filipino, and 500 Chinese adults. Each racial/ethnic group had approximately equal numbers of men and women in three age strata (65–69, 70–74, and 75–79). The Filipino and Chinese samples were smaller than the others because their data were originally intended to be used for pilot study purposes. Due to requirements of the larger patient portal use study of which this survey was one component, the survey sample was restricted to members who had been continuously enrolled in KPNC for at least 2.5 years at the time of the survey.

#### Data collection

Data were collected using a mailed print questionnaire available only in English. Participants were offered a $5 gift card for completing the survey. The survey was first mailed in mid-November 2013, and a follow-up survey was sent in mid-December 2013 to those who had not responded. People who did not respond to either of the first two survey mailings were sent a third, slightly shorter, questionnaire in early February 2014. Participants were told that the survey was being done to help Kaiser Permanente and other organizations learn about seniors’ use of digital tools (like computers, mobile devices, and the internet) and how they prefer to give and get information about their health and health care. A copy of the survey questionnaire is found in Additional file 1.

#### Study variables

##### Sociodemographic and health characteristics

Age, sex, and race/ethnicity were available from member administrative data and used for sample selection, but this information was also requested on the survey to confirm assignment to study subgroups. The survey also asked about level of education, self-rating of health, and ongoing use of at least one prescription medication. The survey did not ask about household income, but for sample description purposes, we estimated sample household income distribution using pooled data from general health surveys conducted with seniors in this same health plan in 2011 and 2014 [33].

##### Access to and use of digital technology

Individuals were asked whether they had easy access to a computer, tablet, and mobile phone, had internet at home, and about their current use of the internet, email, and text messaging. They were also asked whether they had performed a variety of online tasks in the past 12 months, including obtaining information from websites, using search engines, watching online videos, downloading materials and apps, paying for orders/services, completing forms, and using social media.

##### Perceived ability to perform tasks using digital technology

Individuals were asked to indicate whether they could perform a variety of tasks involving use of digital technology, including reaching a website using a URL in printed material, reading health information online, printing information from a website, watching online videos and streamed programs, attending a webinar, listening to and downloading podcasts, using CD and DVD players, and reading print materials on a tablet or e-reader.

##### Interest in using digital technologies to obtain health information and advice

Individuals were asked about their interest in using a variety of digital modalities for obtaining health information, including email-based health coaching, emailed newsletters, reading information on websites, watching online videos and webinars, online interactive programs, interactive computer programs, watching DVDs, podcasts or audio programs, and health-related apps. For comparison purposes, they were also asked about interest in two non-digital modalities, phone-based health counseling and mailed print health newsletters.

#### Data analysis

All analyses were performed using SAS version 9.3 (SAS Institute, Cary, NC 2011) procedures for complex datasets [34] using data weighted to reflect the underlying age x gender x racial/ethnic composition of the study population. Proc Surveymeans was used to produce weighted percentages with 95% confidence intervals. Proc Surveylogistic was used to test whether percentages significantly differed between age cohorts (70–74 and 75–79 vs. 65–69) after adjusting for gender and race/ethnicity, and between racial/ethnic groups (black, Latino, Filipino, and Chinese vs. white) after adjusting for age group and sex. All comparisons between age groups and racial/ethnic groups mentioned in the text are statistically significant with a Wald chi-square value of at least *P* < .05; if an age group or racial/ethnic group is not mentioned in a comparison, it did not significantly differ from the reference group (age 65–69 or white). We did not adjust for multiple comparisons, but we have reported results of all comparisons. In the text, unless otherwise noted, percentages reported as indicating a decline or increase by age refer to, in order, age groups 65–69 (young seniors), 70–74 (middle seniors), and 75–79 (older seniors).

## Results

After excluding ineligibles (bad address, non-member, deceased), the overall survey response rate was 53.5%. Response to the survey was similar across the three age groups (52.1%, 53.9%, and 54.5% for ages 65–69, 70–74, and 75–79, respectively), with no significant difference by sex within age group. Whites and Chinese were more likely to respond than blacks, Latinos, and Filipinos (65.3% and 62.2% vs. 43.9%, 50.5%, and 44.4%, respectively), with no significant difference in response by age or sex within each race/ethnic group.

Characteristics of the weighted study sample are shown in Table 1. Approximately 70% of the seniors had been diagnosed with a chronic cardiovascular condition, and 90% were taking ≥1 one prescription medication for a chronic condition. The sample is predominantly white (79%) and educated beyond high school (64%), with about 40% having a college degree. Nearly 30% of seniors would be considered lower income by community standards (household income ≤$35,000/year). Black and Latino seniors were less likely to be college graduates than white seniors, and nearly one-fourth of Latinos had not graduated from high school compared with approximately 4% of the other groups. Black, Latino, and Filipino seniors were more likely to have a low household income. Compared to the younger group, seniors in the two older groups were less likely to be college graduates and more likely to be low income.

**Table 1:** Characteristics of survey respondents, after weighting, by age group and race/ethnicity

Respondent Characteristics	All	By Age Group			By Race/Ethnicity				
	65–79	65–69	70–74	75–79	White	Black	Latino	Filipino	Chinese
	(*N* = 2602)	(*N* = 841)	(*N* = 878)	(*N* = 883)	(*N* = 849)	(*N* = 567)	(*N* = 653)	(*N* = 219)	(*N* = 314)
	Wtd. %	Wtd. %	Wtd. %	Wtd. %	Wtd. %	Wtd. %	Wtd. %	Wtd. %	Wtd. %
Sociodemographic									
Age									
65–69 yrs.	23.5	n/a	n/a	n/a	23.4	23.6	23.6	25.6	23.1
70–74 yrs.	43.7	n/a	n/a	n/a	43.8	43.3	42.3	45.0	42.0
75–79 yrs.	32.8	n/a	n/a	n/a	32.8	33.1	34.1	29.4	34.9
Sex									
Women	54.1	53.8	53.9	54.5	53.8	56.9	54.8	57.1	48.2
Men	45.9	46.2	46.1	45.5	46.2	43.1	45.2	42.9	51.8
Race/ethnicity									
White non-Hispanic	79.4	79.0	79.6	79.5	n/a	n/a	n/a	n/a	n/a
Black	7.3	7.3	7.3	7.4	n/a	n/a	n/a	n/a	n/a
Hispanic/Latino	5.4	5.4	5.2	5.6	n/a	n/a	n/a	n/a	n/a
Filipino	5.2	5.6	5.3	4.6	n/a	n/a	n/a	n/a	n/a
Chinese	2.7	2.7	2.6	2.9	n/a	n/a	n/a	n/a	n/a
Educational attainment									
Non-high school graduate	5.0	3.2	3.1	8.9^***^	3.9	4.7	22.0^***^	4.7	4.1
High school graduate/GED	21.3	14.5	19.9	28.1	21.0	25.2	31.0	14.1	14.3
Some college	23.8	33.4	36.1	30.8	34.2	45.0	27.8	22.7	24.6
College graduate	39.9	48.9	40.9^*^	32.2^***^	40.9	25.0^***^	19.2^***^	58.4^***^	57.0^***^
Household income^a^									
≤ $25,000	16.2	11.1	17.1^***^	25.7^***^	14.3	25.0^***^	22.1^***^	27.5^***^	13.6
$25,001–$35,000	11.8	9.5	12.6	15.4	11.2	13.7	15.9	14.4	9.2
$35,001–$50,000	16.7	14.9	19.1	17.2	16.3	19.3	19.7	18.2	11.9
$50,000–$80,000	25.3	27.2	24.3	22.8	25.9	21.2	24.9	21.5	25.3
> $80,000	30.0	37.3	26.9^***^	18.9^***^	32.3	20.8^***^	17.4^***^	18.4^***^	40.0
Health									
Self-rated health									
Very good or excellent	43.8	49.7	46.8	35.6^***^	48.3	21.2^***^	28.0^***^	25.9^***^	38.9^e^
Good	38.1	34.8	38.2	40.2	35.9	48.8	39.9	49.7	44.8
Fair or poor	18.1	15.5	15.0	24.2^***^	15.8	30.0^***^	32.1^***^	24.4^***^	16.3
History of diabetes, hypertension, CAD, heart failure, or stroke	71.7	62.1	70.3^g^	80.6^***^	69.0	87.4^***^	77.8^***^	86.5^***^	70.5
Takes medication for ≥1 chronic condition	90.5	87.2	90.3	93.3^**^	89.9	95.4^***^	90.7	94.9^*^	86.8
Access to digital technology									
Desktop, laptop, or netbook computer	79.5	90.4	80.0^***^	71.2^***^	83.5	69.1^***^	61.1^***^	53.1^***^	79.8
Tablet	25.1	34.3	27.4^*^	15.6^***^	27.1	16.0^***^	12.6^***^	20.1^*^	28.3
Smartphone	31.2	43.4	33.9^**^	18.7^***^	32.8	30.5	22.0^***^	19.6^***^	26.6^***^
Home internet	83.8	91.3	85.5^**^	76.2^***^	87.4	71.9^***^	68.4^***^	61.0^***^	84.8

Cell percentages are based on weighted data for everyone in that age or racial/ethnic group. Ns at top of columns are the unweighted number of respondents in that group

*White* Non-Hispanic white, *GED* General Educational Development, *CAD* coronary artery disease

^*^Significant difference *(P < .05)* after controlling for race/ethnicity and sex (age group comparisons) or age group and sex (racial/ethnic group comparisons)

^**^Significant difference *(P < .01)* after controlling for race/ethnicity and sex (age group comparisons) or age group and sex (racial/ethnic group comparisons)

^***^Significant difference *(P < .001)* after controlling for race/ethnicity and sex (age group comparisons) or age group and sex (racial/ethnic group comparisons)

^a^The survey questionnaire did not include a question about income. These statistics are based on pooled data from 2011 and 2014 health surveys of the same health plan membership weighted to the 2014 age-sex composition of the membership. In the San Francisco Bay Area, a household income ≤$35,000 qualifies an individual for low income housing

### Access to digital devices and the internet

Over three-fourths of 65–79 year olds reported easy access to a desktop, laptop, or netbook computer, and 25% had a tablet. However, easy access to these devices declined by age and was lower among blacks, Latinos, and Filipinos than whites (Table 1). While approximately three-fourths of 65–79 year olds had a mobile phone, less than one-third had a smartphone. Smartphone ownership declined with age and was lower among Latinos and Filipinos than whites. Most (> 90%) seniors who owned a tablet or smartphone also had access to a computer, and while 44% of internet-using seniors sometimes accessed the internet using a mobile device (27% tablet, 28% smartphone), most (96%) also did so using a desktop or laptop computer. Over 80% of seniors had access to the internet at home, and nearly all (> 97%) seniors who used the internet indicated being able to do so from home.

### Ability to use the internet, email, and text messaging

Approximately 80% of the seniors were able to go online by themselves or with help from someone to obtain health information from websites or to communicate with others (Table 2). Seniors in the 65–69 year-old age group were more likely to be internet users than those in the two older age groups, and white and Chinese seniors were more likely to be users than black, Latino, and Filipino seniors. Approximately 10% of internet users required help. The percentages of seniors able to send and receive email (not shown) were virtually the same across age and racial/ethnic groups as those for ability to use the internet. Less than half (47%) of seniors reported being able to receive and send text messages. While ability to use text messaging did not vary greatly by race/ethnicity, a decline by age was observed even among those who had mobile phones (71%, 63%, 47%).

**Table 2:** Seniors’ perceptions of their ability to perform activities using digital technologies on their own or with a little help

Digital Technology Activities	All	By Age Group			By Race/Ethnicity				
	65–79	65–69	70–74	75–79	White	Black	Latino	Filipino	Chinese
	(*N* = 2429)	(*N* = 776)	(*N* = 819)	(*N* = 834)	(*N* = 835)	(*N* = 489)	(*N* = 612)	(*N* = 191)	(*N* = 302)
Use the internet to get information from websites or communicate with others^a,b^,%	79.4	88.9	81.5^***^	68.7^***^	83.9	64.4^***^	58.2^***^	53.3^***^	79.2
Receive and send text messages,%	47.2	61.5	51.4^**^	31.4^***^	47.4	53.6^*^	41.0^*^	45.1	40.5
Go to a website to get information or forms using a URL (website address) given orally or in a letter ^a^,%	60.2	75.3	61.8^***^	47.4^***^	63.3	49.6^***^	42.6^***^	42.9^***^	65.2
Print information or forms from a website ^a^,%	70.4	81.7	72.3^***^	59.9^***^	74.0	58.1^***^	50.8^***^	50.8^***^	74.3
Read health information online on a website like kp.org,%	65.1	75.6	68.0^*^	53.7^***^	70.2	46.5^***^	42.8^***^	38.8^***^	61.0^**^
Watch a video online	50.9	64.8	53.5^***^	37.3 ^***^	55.3	36.6^***^	31.4^***^	26.6^***^	45.2^**^
Watch movies and programs sent to TV or computer over the internet (e.g., Netflix),%	34.7	43.9	36.3^*^	25.9^***^	36.9	29.6^*^	26.3^***^	19.8^***^	27.9^**^
Watch or listen to a webinar (live talk given ona website) using a computer or smartphone,%	23.4	34.4	26.0^**^	12.0^***^	25.5	18.5^**^	14.8^***^	10.9^***^	14.2^***^
Watch a DVD on TV or computer,%	76.4	81.9	79.0	68.9^***^	77.8	75.6	75.6	58.9^***^	71.4^*^
Listen to an audio CD on a CD player,%	60.0	66.7	62.8	51.3^***^	63.0	55.1^**^	51.3^***^	36.3^***^	45.5^***^
Listen to a podcast or music on an iPod, MP3 player, or computer,%	26.7	38.0	27.6^**^	17.4^***^	29.0	21.8^**^	19.2^***^	10.3^***^	19.4^**^
Use an interactive CD-ROM or DVD program on a computer,%	37.0	49.4	37.3^***^	27.8^***^	39.8	32.0^**^	24.6^***^	17.6^***^	30.8^**^
Download a podcast or music from a website to play on an iPod, MP3 player, or computer,%	19.8	32.6	19.7^***^	10.6^***^	21.7	14.9^**^	11.9^***^	8.7^***^	13.4^**^
Read books or other print material on a tablet (e.g., iPad) or e-reader (e.g., Kindle, Nook),%	35.4	44.9	36.0^**^	28.0^***^	37.1	30.9^*^	24.9^***^	30.0	31.6

Cell percentages are based on weighted data for everyone in that age or racial/ethnic group. Ns at top of columns are the unweighted number of respondents in that group. Statistical testing compared ages 70–74 and 75–79 with ages 65–69 and blacks, Latinos, Filipinos, and Chinese with non-Hispanic whites. Unless otherwise noted, percentages are based on data from the 93% of respondents who completed the long form of the questionnaire used for the first 2 mailings

*White* Non-Hispanic white

^*^Significant difference *(P < .05)* after controlling for race/ethnicity and sex (age group comparisons) or age group and sex (racial/ethnic group  comparisons)

^**^Significant difference *(P < .01)* after controlling for race/ethnicity and sex (age group comparisons) or age group and sex (racial/ethnic group comparisons)

^***^Significant difference *(P < .001)* after controlling for race/ethnicity and sex (age group comparisons) or age group and sex (racial/ethnic group comparisons)

^a^Based on the full respondent sample (see Table 1 for column Ns).

^b^69.4% were able to use the internet by themselves; by age group, 80.7%, 70.8%, and 59.4%; by race/ethnicity, 74.4%, 51.8%, 48.05, 39.3%, and 69.4%, respectively

### Ability to use eHealth technologies to obtain health information and advice

Table 2 shows that perceived ability to use eHealth technologies varies substantially across modalities. Approximately three-quarters of seniors said they could watch a DVD on television or computer, 60% thought they would be able to listen to an audio CD, and 27% that they would be able to listen to podcasts or audio programs on an iPod, MP3 player, or computer. Black, Latino, Filipino, and Chinese were less likely than white seniors to indicate ability to use these types of devices, and declines by age group were observed. Approximately one-third of seniors could read books or other materials on a tablet or e-reader and use an interactive CD-ROM or DVD program on a computer, but these capabilities also declined with age.

Approximately 60% of seniors reported that they could read health information online, 50% could watch online videos, 35% could watch streamed programs, and 23% could watch or listen to a webinar. Ability to perform these tasks decreased with age and was less prevalent among black, Latino, Filipino, and Chinese seniors than whites.

### Recent experience performing online tasks

Seniors were asked to indicate whether they had gone online in the prior 12 months to perform thirteen tasks on their own or with a little help. The results are found in Table 3.

**Table 3:** Tasks involving digital technology seniors did in the past 12 months on their own or with a little help

Digital Technology Tasks	All	By Age Group			By Race/Ethnicity				
	65–79	65–69	70–74	75–79	White	Black	Latino	Filipino	Chinese
	(N = 2602)	(N = 841)	(N = 878)	(N = 883)	(N = 849)	(N = 567)	(N = 653)	(N = 219)	(N = 314)
Searched for information on the internet about any type of product, service, or problem using Google/other search engine,%	72.2	84.4	72.9^***^	62.6^***^	76.7	55.0^***^	54.7^***^	47.1^***^	70.8
Got information about a health topic from any website,%	62.9	75.7	64.2^***^	52.0^***^	66.6	48.0^***^	48.2^***^	41.8^***^	63.4
Downloaded a printed a form or other document from a website,%	61.2	73.4	63.3^**^	49.7^***^	65.5	44.2^***^	43.7^***^	37.7^***^	61.8
Clicked on a website link that opened a document or took you to another part of the website or another website,%	54.3	73.5	54.9^***^	39.6^***^	58.0	40.3^***^	37.0^***^	35.1^***^	51.8
Filled out an online form or questionnaire and then submitted it,%	52.4	65.2	55.0^**^	39.8^***^	56.0	40.3^***^	33.6^***^	32.3^***^	54.3
Used online chat to get information or help from a company’s website ^a^,%	20.9	28.6	23.3	12.4^***^	21.6	18.9	15.6^**^	18.5	22.2
Used Skype/video chat to view the person youwere talking to on screen,%	20.3	24.9	22.8	13.5^***^	20.7	15.8^*^	17.5	18.9	26.4^*^
Paid a bill or ordered any product or service online using a credit card,%	62.5	72.9	65.9^*^	50.5^***^	66.9	45.5^***^	44.7^***^	39.0^***^	59.3^*^
Paid a bill or ordered any product or service online using PayPal^a^,%	28.1	38.8	28.6^**^	19.8^***^	29.2	25.1	22.6^**^	20.0^*^	31.5
Looked at someone’s or a company’s Facebook page ^a^,%	29.8	38.7	31.2^*^	21.6^***^	31.7	21.9^***^	22.5^***^	21.1^**^	26.0
Used Twitter ^a^,%	7.6	10.4	8.4	4.4^***^	7.2	7.1	7.7	11.9^*^	10.9
Downloaded an app from a website (Apple app store, Google Play, etc.) ^a^,%	33.8	45.5	36.2^**^	22.1^***^	35.1	28.5^*^	27.1^**^	27.2^*^	35.1
Used a health-related app (e.g., to monitor blood pressure, sleep, food eaten, exercise, etc.) on a smartphone or iPad/other tablet computer,%	11.6	17.8	11.6^*^	7.0^***^	11.1	12.6	10.9	16.2	14.1

Cell percentages are based on weighted data for everyone in that age or racial/ethnic group. Ns at top of columns are the unweighted number of respondents in that group. Statistical testing compared ages 70–74 and 75–79 with ages 65–69 and blacks, Latinos, Filipinos, and Chinese with non-Hispanic whites. Unless otherwise noted, percentages are based on data from the full respondent sample

*White* Non-Hispanic white

^*^Significant difference *(P < .05)* after controlling for race/ethnicity and sex (age group comparisons) or age group and sex (racial/ethnic group comparisons)

^**^Significant difference *(P < .01)* after controlling for race/ethnicity and sex (age group comparisons) or age group and sex (racial/ethnic group comparisons)

^***^Significant difference *(P < .001)* after controlling for race/ethnicity and sex (age group comparisons) or age group and sex (racial/ethnic group comparisons)

^a^Based on the respondents who completed the longer form of the questionnaire (see Table 2 for column Ns)

We categorized these tasks as (a) obtaining information or forms from an online source; (b) providing or requesting information online, including communicating with others; (c) paying for services or products online; (d) using social media; and (e) using apps.

#### Obtained information online

Over 70% of seniors had used a search engine to do a web search for information about a product, service, or problem, and 63% had obtained health information from a website. Approximately 54% had clicked on a web link to open a document or move to a different web location, and 60% had downloaded or printed material from a website. Blacks, Latinos, Filipinos, and adults in the two older groups were less likely than whites and younger seniors, respectively, to have performed these tasks.

#### Provided or requested information online

Approximately half of seniors had filled out an online form or questionnaire and then submitted it online. Around one-fifth had used online chat to get information or help from a company’s website, and a similar percentage had used Skype or another video chat service. Blacks, Latinos, and Filipinos were less likely than whites to have performed these tasks in the past year, as were those aged 75–79 compared to 65–69 year olds.

#### Paid for products or services online

Sixty-two percent of seniors had paid a bill or ordered a product or service online using a credit card, and 28% had paid for a product or service online using PayPal. Blacks, Latinos, and Filipinos were less likely than whites to have paid for something online, and 75–79 year olds were less likely than 65–69 year olds to have done so.

#### Used social media

Nearly 30% of seniors had looked at someone’s or a company’s Facebook page. While nearly 40% of those aged 65–69 had used Facebook, this dropped to 31% of 70–74 year olds and 18% of 75–79 year olds, and racial/ethnic group differences (lower percentages of blacks, Latinos, and Filipinos compared to whites) were observed across all age groups. Twitter had been used by only about 8% of seniors overall and 10% of those aged 65–69.

#### Used apps

Approximately 12% of seniors said they had used a health-related app on a smartphone or tablet computer in the past 12 months, but usage was lower among the older groups.

One-third of seniors (but 60% of those with a smartphone or tablet) said they had downloaded any type of app from a website at least once, but again, this declined with age, and blacks, Latinos, and Filipinos were less likely than whites to have done so.

### Willingness to use eHealth modalities to obtain health information and advice

Seniors were asked to indicate which of a variety of primarily digital modalities they would like to use to get health information and advice in addition to directly from their doctor and other clinicians. Results are shown in Table 4. The method most frequently indicated across all age and racial/ethnic groups was having telephone appointments with a health coach. Over half of seniors indicated a preference for this type of interpersonal communication, with ≥70% of blacks, Latinos, and Filipinos preferring it as compared to 50% of whites and Chinese. The percentages expressing interest in getting health coaching by email were significantly lower (29%) but still substantially higher than most of the other eHIA modalities listed.

**Table 4:** Percentages of seniors interested in getting health information and advice using eHealth modalities^a^

eHealth information modality	All	By Age Group			By Race/Ethnicity				
	65–79	65–69	70–74	75–79	White	Black	Latino	Filipino	Chinese
	(N = 2602)	(n = 841)	(n = 878)	(n = 883)	(n = 849)	(n = 567)	(n = 653)	(n = 219)	(n = 314)
Phone calls with a Health Coach,%	54.0	54.6	50.4	58.6	50.2	70.2^***^	71.7^***^	73.6^***^	52.2
Help from a Health Coach by email *(sent* via *patient portal* vs. *regular email not specified in question),%*	28.7	33.9	28.7	25.0^**^	29.6	22.4^***^	26.2	21.7^*^	35.6
Read about health topics online on the health plan/other website,%	35.4	42.4	38.4	26.4^***^	39.1	22.4^***^	20.5^***^	15.9^***^	28.3^**^
Watch a video online on the health plan website or site like YouTube,%	24.5	27.4	27.9	18.0^**^	26.7	17.5^***^	15.2^***^	11.6^***^	24.3
Watch a DVD at home,%	16.1	15.5	16.4	16.1	15.7	20.7^*^	16.5	12.5	21.9^*^
Watch a video sent over the internet to a TV, DVD player, or computer by Netflix/Other,%	9.0	9.4	10.0	7.4	9.5	8.2	6.5	6.6	7.5
Use an interactive DVD or CD-ROM on a computer,%	7.4	10.8	7.2	5.1^**^	7.9	8.0	3.9^**^	3.5^*^	5.2
Watch live or recorded online webinar talks/presentations on a website like kp.org,%	14.3	15.0	17.3	9.8^*^	15.5	11.6^*^	9.4^***^	6.7^**^	8.1^**^
Use online interactive programs (read information, answer questions, get feedback),%	16.3	20.7	18.6	9.9^***^	18.0	11.4^***^	9.3^***^	5.3^***^	13.8
Listen to an audio podcast or other audio program on a computer, iPod, or MP3 player,%	5.4	4.9	6.8	4.0	5.9	4.6	3.6	2.7	3.2
Use a health-related app on a smartphone or tablet (e.g., iPad),%	7.2	10.8	7.4	4.3^***^	7.6	6.5	6.2	5.4	2.7^**^
Those who have a mobile device,%	17.0	18.4	17.0	14.8	17.0	17.0	20.9	19.0	6.5^**^
Join an online chat room or online community for members with similar health problems,%	4.7	6.5	4.9	3.0^*^	4.7	5.7	3.2	5.2	2.7

Cell percentages are based on weighted data for everyone in that age or racial/ethnic group. Ns at top of columns are the unweighted number of respondents in that group except when analyses are restricted to a subset of that group. Statistical testing compared ages 70–74 and 75–79 with ages 65–69 and blacks, Latinos, Filipinos, and Chinese with non-Hispanic whites. Unless otherwise noted, percentages are based on data from the full respondent sample

*White* Non-Hispanic white

^*^Significant difference *(P < .05)* after controlling for race/ethnicity and sex (age group comparisons) or age group and sex (racial/ethnic group comparisons)

^**^Significant difference *(P < .01)* after controlling for race/ethnicity and sex (age group comparisons) or age group and sex (racial/ethnic group comparisons)

^***^Significant difference *(P < .001)* after controlling for race/ethnicity and sex (age group comparisons) or age group and sex (racial/ethnic group comparisons)

^a^Based on the respondents who completed the longer form of the questionnaire (see Table 2 for column Ns)

Approximately one-third of seniors were interested in reading about health topics online. Those aged 75–79 were less likely than those aged 65–74 to be interested in this method, and blacks, Latinos, Filipinos, and Chinese were less interested than whites.

Of three methods for watching videos on health topics, watching online videos appeared to be somewhat more appealing to seniors (24%) than watching DVDs at home (16%), with streamed videos (9%) receiving the least interest. The higher percentage interested in watching online health videos versus DVDs was driven mostly by whites and Chinese aged 65–74 (29% and 32%, respectively). Overall, preference for watching health videos online was lower among black, Latino, and Filipino seniors than whites, and among those aged 75–79 compared to 65–74 year olds (18% versus 27%).

Approximately 15% of seniors were interested in watching live or recorded online webinar talks/presentations or using online interactive programs. Again, blacks, Latinos, and Filipinos and older seniors were less likely than non-Hispanic whites and seniors aged 65–74, respectively, to prefer these modalities. Seniors expressed little interest in listening to podcasts or joining an online chat room or online community for members with similar health problems. About 7% of seniors overall and approximately 10% of those aged 65–69 were interested in using a health-related app on a smartphone or tablet, although this increased to 17% when restricted to people who currently have a smartphone or tablet. Interest in using health-related apps was lowest in the oldest age group (4%), although there was no significant age difference among those who had a mobile device. Overall and among mobile device owners, Chinese seniors showed the lowest level of interest in using health apps among all racial/ethnic groups.

### Preferences for mailed versus emailed health newsletters

Seniors were asked to indicate how they would prefer and be willing to receive health newsletters. Overall, larger percentages of seniors preferred to receive print newsletters mailed to their home rather than newsletters delivered via email (59% vs. 38%) (Table 5). Seniors of color and those aged 75–79 were more likely to prefer mailed newsletters and less likely to prefer emailed newsletters than white and 65–69 year olds, respectively. As reported in an earlier publication [19], seniors were more likely to be willing to and to prefer to receive emailed newsletters if the newsletter was in the body of the email (39.2% and 21.6%, respectively) than if it was an attached pdf (23.8% and 10.4%, respectively) or linked to from the email (24.0% and 9.5%, respectively).

**Table 5:** Methods seniors are willing to use and would prefer for receiving health newsletters

Health newsletter modality	All	By Age Group			By Race/Ethnicity				
	65–79	65–69	70–74	75–79	White	Black	Latino	Filipino	Chinese
	(*N* = 2377)	(*n* = 769)	(*n* = 790)	(*n* = 818)	(*n* = 815)	(*n* = 480)	(*n* = 594)	(*n* = 187)	(*n* = 301)
Get a print newsletter by regular mail									
Willing to use this method,%	65.9	62.0	60.5	75.8^***^	62.7	80.3^***^	77.8^***^	80.8^***^	68.1
Most prefers this method,%	58.7	51.9	52.3	71.7^***^	54.1	76.4^***^	76.6^***^	82.2^***^	65.1^**^
Get the newsletter in the body of the email, in an attached pdf, or via a link from the email									
Willing to use this method,%	54.5	62.5	59.3	42.2^***^	59.3	37.2^***^	34.4^***^	30.0^***^	48.2^**^
Most prefers this method,%	38.4	44.0	44.2	26.6^***^	42.7	21.2^***^	22.1^***^	16.5^***^	32.0^**^

Cell percentages are based on weighted data for everyone in that age or racial/ethnic group; Ns at top of columns are the unweighted number of respondents in that group. Cell percentages in the “All” and “Age Group” columns are for all adults in the age group, not just those in the five racial/ethnic groups. Age group comparisons control for race/ethnicity and sex, and racial/ethnic group comparisons control for age group and sex. Results are based on data obtained from respondents who completed the longer form of the questionnaire (see Table 2 for column Ns)

*White* Non-Hispanic white

^**^Significant difference *(P < .01)* after controlling for race/ethnicity and sex (age group comparisons) or age group and sex (racial/ethnic group comparisons)

^***^Significant difference *(P < .001)* after controlling for race/ethnicity and sex (age group comparisons) or age group and sex (racial/ethnic group comparisons)

### Differences in preference for going online for health information are not solely explained by access to ability to use the internet

We examined interest in obtaining information and advice using eHealth modalities among the 80% of seniors who currently use the internet by themselves or with help from someone (Table 6). It should be noted that restricting to internet users at the outset disproportionately excludes older (30.3% vs. 11.1%, 18.5%, respectively) and black, Latino, and Filipino seniors (35.6%, 41.8%, 46.7%, respectively, vs. 16.1% of whites and 20.8% of Chinese). While interest in using these eHealth education modalities is higher among internet users than the full population, we still find significant age group and racial/ethnic disparities in interest in reading about health topics online and getting health newsletters by email, and across all age and racial/ethnic groups, these seniors indicated more interest in getting health advice by talking with someone rather than through email exchanges.

**Table 6:** Percentages of current internet users interested in obtaining health information and advice online, by email, or by phone

eHealth information modality	All	By Age Group			By Race/Ethnicity				
	65–79	65–69	70–74	75–79	White	Black	Latino	Filipino	Chinese
	(*N* = 1938)	(*n* = 728)	(*n* = 656)	(*n* = 564)	(*n* = 732)	(*n* = 404)	(*n* = 425)	(*n* = 132)	(*n* = 235)
Read about health topics online on a website*,%*	44.3	47.7	46.5	37.8^**^	46.4	34.0^***^	33.6^***^	29.8^***^	35.0^**^
Watch an online video on kp.org or another website like YouTube*,%*	30.4	30.7	33.4	25.7	31.4	26.8	24.9^*^	20.2^*^	30.3
Use online interactive programs (read information, answer questions, get feedback),%	20.4	23.2	22.6	14.2^**^	21.4	17.1	15.2^*^	8.4^**^	17.4
Emailed health newsletter^a^,%	68.1	70.7	71.7	60.1^**^	70.4	57.6^***^	56.8^***^	50.5^***^	59.3^**^
Phone calls with a Health Coach,%	49.5	50.6	47.0	52.2	47.5	60.4^***^	64.7^***^	63.1^**^	47.9
Help from a Health Coach by email*,%*	34.9	37.5	33.8	34.1	34.5	32.4	38.8	37.2	42.4^*^

Cell percentages are based on weighted data for all adult who can use the internet alone or with help in that age or racial/ethnic group; Ns at top of columns are the unweighted number of respondents in that group. Cell percentages in the “All” and “Age Group” columns are for all adults in the age group, not just those in the five racial/ethnic groups. Age group comparisons control for race/ethnicity and sex, and racial/ethnic group comparisons control for age group and sex

*White* Non-Hispanic white

^*^Significant difference *(P < .05)* after controlling for race/ethnicity and sex (age group comparisons) or age group and sex (racial/ethnic group comparisons)

^**^Significant difference *(P < .01)* after controlling for race/ethnicity and sex (age group comparisons) or age group and sex (racial/ethnic group comparisons)

^***^Significant difference *(P < .001)* after controlling for race/ethnicity and sex (age group comparisons) or age group and sex (racial/ethnic group comparisons)

^a^Percentages reported are those who are willing to receive health newsletters by email. For all age and racial/ethnic groups, the percentages who *preferred* getting newsletters by email were approximately 20 percentage points less. Results are based on data from respondents who completed the longer form of the questionnaire (see Table 2 for column Ns)

## Discussion

In an earlier article based on the same study, we reported that there were significant age and racial/ethnic disparities in older adults’ engagement with the health plan’s patient portal and preferences for exchange of health information using eHealth technologies [17]. In this current article, we showed that in addition to disparities in access to digital technology, older seniors and black, Latino, and Filipino seniors have less experience performing a variety of online tasks relevant to engagement with digital platforms for health information, purchase of self-care products and services, and monitoring of behaviors and health measures using health apps. They are also less likely to be interested in using online health education resources. This suggests that without encouragement and help, the segments of the older adult population who are already more vulnerable to chronic health problems and health care access barriers are likely not going to take advantage of web-based health information and health education services, other types of health-related digital tools and mobile apps, and opportunities to engage in one-way or bidirectional communications related to their health using secure messaging, text messaging, and interactive online sessions with their health care providers.

Specifically, we found that black, Latino, Filipino, and older seniors (75–79 year olds) were less likely than white and Chinese and younger (65–69 year olds) seniors, respectively, to have the digital tools (devices and internet access), skills, and experience to take advantage of new web- and mobile-based modalities for communicating (regular email and secure messages sent through a patient portal or using an app) and obtaining health-related information and advice. Black, Latino, Filipino, and older seniors were also less likely to have gone online in the previous 12 months to obtain information or forms, download or print documents, provide or request information, complete a web-based form or questionnaire, pay for products and services, and use Facebook. Disparities were not seen in use of Twitter and health-related apps, but overall usage of these technologies across all groups was under 15%. The existence of digital divides by age group and race/ethnicity in the adult U.S. population has been well-documented, as have digital divides in patient portal use [35–39]. However, we believe that our study is among the first to focus on age group and racial/ethnic digital divides among older adults in access to a variety of digital technologies, experience and skills in performing a wide variety of tasks using these technologies that are relevant to health education and self-care, and willingness to use different digital modalities for health education and health promotion. Additionally, our study contributes new information about use of digital technologies and digital resources for health-related purposes by Filipino and Chinese American seniors.

Across all age and racial/ethnic groups, but especially among older seniors and blacks, Latinos, and Filipinos, we observed much greater interest in obtaining health information and advice by talking with someone over the telephone and by receiving newsletters in the mail than in getting health information and advice via emails or by using other eHIA modalities. This was observed for seniors overall and for those who are able to use the internet alone or with help. Based on focus groups conducted with black and Latino members from the same health plan, Lyles and colleagues concluded that many individuals did not feel that they could sufficiently understand information provided through online communication alone [40]. Blacks, Latinos, Filipinos, and older seniors are also less likely to be able to or willing to read about or watch online videos about health than whites, Chinese, and younger seniors, and also to be less interested in receiving health newsletters by email than through regular mail. Less than 10% of all seniors and under 20% of those who use a mobile device are interested in using health-related apps, and only around 5% are interested in listening to podcasts or joining online chat rooms. It is important to consider racial/ethnic and age-related differences in preferences when planning and implementing approaches to patient education and out-of-clinic monitoring of behaviors and vital signs mHealth monitoring approaches for a diverse older patient population.

Additionally, it is likely going to be more feasible for healthcare and community organizations to mount efforts to improve eHealth skills and change preferences among those seniors who already have easy access to digital devices and the internet than to address financial and other barriers to patients’ access to the internet and digital devices. In-person training programs, which can be held in libraries and community centers, have been shown to increase seniors’ use of the internet to obtain health information and advice [41, 42]. Instructions for how to use search engines, download and print materials, complete online forms, and use a health plan’s website and patient portal should be available as downloadable pdfs or hardcopy print materials, not just web-based tutorials that are difficult to refer to while trying to perform the tasks. Additionally, having a toll-free line that people can easily call for assistance rather than solely providing web-based instructions and web-based coaching options can help reduce the frustration often associated with undertaking a new or infrequently used action.

When planning health information and patient education modalities for a multi-cultural and multi-generational patient population, it is important to take into account digital divides and make sure that traditional information channels remain available and publicized to those who may need them. Web-based patient education resources, apps, and other eHealth modalities should supplement, not supplant, the traditional modes of personal communication and support valued by many seniors [43]. At the individual patient level, as part of providing good patient-centered care, it is important for providers to assess rather than assume digital access, eHealth skills, capacity to download and print materials, and preferred methods for receiving information prior to directing their patients to online and mHealth resources.

### Strengths of this study

Our study had several strengths. We examined a broad range of factors relevant to access and potential use of eHealth education modalities by older adults, including access to and ability to use digital devices and the internet, and recent experience with, perceived ability to perform, and preferences for performing a wide variety of web-based tasks. Our age and race/ethnically stratified survey sample made it possible to show that racial/ethnic and age-related digital divides with regard to use of and interest in using digital resources for obtaining health information and advice persist even among those who are currently using the internet on their own or with someone’s help. Finally, our inclusion of Filipino and Chinese American subgroups in the survey enabled us to provide descriptive data about “digital readiness” and preferences of these seniors for obtaining health information and advice using digital technologies and to show that combining these two ethnic groups into a broad “Asian” category might yield misleading information when planning patient-facing eHealth services.

### Limitations of this study

A major limitation of the study is that we conducted the survey only with members of Kaiser Permanente’s integrated healthcare delivery program in Northern California region (KPNC). The health plan and its medical group have increasingly promoted use of the comprehensive online health information and patient education/health promotion resources available on its kp.org website, many of which do not require members to sign onto the patient portal to access. Additionally, most KPNC seniors reside in urban and suburban areas with strong penetration of high speed broadband service for homes as well as free internet access at most libraries and senior centers. This may limit the generalizability of our study results to other parts of the country where the internet is not as available to seniors due to cost or infrastructure.

A second limitation of the study is the response rate, and especially the lower response by black, Latino, and Filipino seniors as compared with non-Hispanic whites and Chinese. This impacted our statistical power to detect racial/ethnic differences within age groups, and again, may limit the generalizability. Additionally, the smaller numbers of Filipino and Chinese seniors in the sample limited the statistical power of racial/ethnic comparisons.

## Conclusions

There are significant age and racial/ethnic eHealth digital divides among older adults with respect to ownership of digital devices, skills and experience with performing digital tasks, and preferences for using online resources to obtain health information and advice. These eHealth disparities could increase disparities in healthcare access and health outcomes if already vulnerable seniors have difficulty obtaining health information and advice, are not able to use digital monitoring tools recommended by their clinicians, and experience higher out-of-pocket costs for medical supplies than those able to make purchases online. If health plans want to bridge the digital divide in access to and use of eHealth information and education resources and mHealth monitoring devices by seniors, those on the other side of the digital divide will need ongoing encouragement from their healthcare providers to try these new resources. They will also need help through multiple modalities with learning and becoming comfortable with eHealth technology. Finally, as part of good patient-centered care, it will be important for health plans to continue to offer more traditional modalities of delivering health information and advice alongside digital approaches if that is what the patient prefers.

## Additional file

Additional file 1: Survey questionnaire. (PDF 316 kb)

## Abbreviations

CD: Compact disk
CD-ROM: Compact disk with read only memory
CI: Confidence interval
DVD: Digital Versatile Disk (formerly called Digital Video Disk)
eHIA: Use of eHealth modalities for health information and advice
KPNC: Kaiser Permanente Northern California
SAS: Statistical Analysis System for personal computers
U.S: United States
